# Implementing a no-drain policy for extraperitoneal colorectal anastomosis in a real-life setting: analysis of outcomes and surgeons’ adherence

**DOI:** 10.1007/s00384-024-04681-0

**Published:** 2024-07-15

**Authors:** Jacopo Crippa, Antonio Luberto, Carmelo Magistro, Michele Carvello, Pietro Carnevali, Annalisa Maroli, Giovanni Carlo Ferrari, Antonino Spinelli

**Affiliations:** 1 Department of Biomedical Sciences, Humanitas University, Via Rita Levi Montalcini 4 Pieve Emanuele, 20072 Milan, Italy; 2 Division of Colon and Rectal Surgery, IRCCS Humanitas Research Hospital, Via Alessandro Manzoni 56 Rozzano, 20089 Milan, Italy; 3 Division of Minimally Invasive Surgical Oncology, ASST Grande Ospedale Metropolitano Niguarda, Piazza Ospedale Maggiore 3, 20162 Milan, Italy

**Keywords:** No-drain policy, Anastomotic drain, Total mesorectal excision

## Abstract

**Aim:**

Recent evidence has questioned the usefulness of anastomotic drain (AD) after low anterior resection (LAR). However, the implementation and adoption of a no-drain policy are still poor. This study aims to assess the clinical outcomes of the implementation of a no-drain policy for rectal cancer surgery into a real-life setting and the adherence of the surgeons to such policy.

**Method:**

A retrospective analysis was conducted on patients who underwent elective minimally invasive LAR between January 2015 and December 2019 at two tertiary referral centers. In 2017, both centers implemented a policy aimed at reducing the use of AD. Patients were retrospectively categorized into two groups: the drain policy (DP) group, comprising patients treated before 2017, and the no-drain policy (NDP) group, consisting of patients treated from 2017 onwards. The endpoints were the rate of anastomotic leak (AL) and of related interventions.

**Results:**

Among the 272 patients included, 188 (69.1%) were in the NDP group, and 84 (30.9%) were in the DP group. Baseline characteristics were similar between the two groups. AL rate was 11.2% in the NDP group compared to 10.7% in the DP group (*p* = 1.000), and the AL grade distribution (grade A, 19.1% (4/21) vs 28.6% (2/9); grade B, 28.6% (6/21) vs 11.1% (1/9); grade C, 52.4% (11/21) vs 66.7% (6/9), *p* = 0.759) did not significantly differ between the groups. All patients with symptomatic AL and AD underwent surgical treatment for the leak, while those with symptomatic AL in the NPD group were managed with surgery (66.7%), endoscopic (19.0%), or percutaneous (14.3%) interventions. Postoperative outcomes were similar between the groups. Three years after implementing the no-drain policy, AD was utilized in only 16.5% of cases, compared to 76.2% at the study’s outset.

**Conclusion:**

The introduction of a no-drain policy received a good adoption rate and did not affect negatively the surgical outcomes.

**Supplementary Information:**

The online version contains supplementary material available at 10.1007/s00384-024-04681-0.

## Introduction

Rectal cancer treatment has undergone remarkable evolution in recent decades [1]. The introduction of total mesorectal excision (TME) and later neoadjuvant chemo-radiotherapy (nCRT) has led to improved oncological outcomes and reduced the incidence of abdominoperineal excision of the rectum [2, 3]. Additionally, minimally invasive surgical techniques, including laparoscopic and robotic procedures, along with modern perioperative protocols, have minimized the overall impact of surgery [4, 5]. However, despite significant breakthroughs in both clinical and oncological fields, anastomotic leak (AL) remains the most dramatic complication in rectal surgery, often associated with high morbidity and mortality [6, 7]. The reported incidence of AL after low anterior resection (LAR) ranges from 3 to 27% in multiple series [8, 9]. A defunctioning stoma may reduce the morbidity of AL and, ultimately, decrease the need for reinterventions [10]. The placing of anastomotic drain (AD) has been traditionally employed as a strategy to detect potential anastomotic leaks and prevent pelvic and abdominal sepsis by draining fecal or purulent discharge. Additionally, the rationale behind AD placement is to minimize postoperative fluid accumulation at the anastomotic site, thereby reducing the risk of infection [10, 11].

While some randomized controlled trials (RCTs) have suggested a potential benefit of AD in reducing the rate of AL [12, 13], subsequent meta-analyses have not provided conclusive evidence regarding the role of AD [10, 14, 15]. Indeed, a Cochrane review [16] highlighted the insufficient evidence to support the use of AD after elective colorectal surgery.

Although most recent evidence questions the usefulness of AD, the debate remains ongoing [17, 18]. Additionally, data on the application and progressive implementation of such policy in real-life settings are poor. Similarly, the lack of strong evidence may reduce surgeons’ adherence to a consistent no-drain policy adoption.

For these reasons, we have undertaken the present study to assess the clinical outomes of a no-drain policy for patients undergoing LAR for rectal cancer and the surgeons’ adherence during the implementation process.

## Methods

A retrospective analysis was conducted on all consecutive patients who underwent elective LAR between February 2015 and December 2019 at two tertiary referral colorectal centers: Humanitas Research Hospital, Rozzano (MI), Italy, and Niguarda Hospital, Milan, Italy. This study adhered to the ethical guidelines of the Declaration of Helsinki. Written informed consent was obtained at surgical enrollment for anonymized patient information to be published in this article. Ethical committee approval was obtained by Comitato Etico indipendente IRCCS Istituto Clinico Humanitas with protocol number 202026281478. The manuscript adheres to the STROBE guidelines for reporting observational studies [19]. This research did not receive any specific grant from funding agencies in the public, commercial, or not-for-profit sectors.

In 2017, both departments implemented a no AD policy. After reviewing the current evidence on drain placement, the chiefs of the respective divisions, during routine meetings, encouraged surgeons to consider avoiding the use of AD (Penrose or Jackson-Pratt drains) as a standard practice. However, the decision remained at the discretion of the individual surgeon.

Patients were divided into two groups: a drain policy (DP) group consisting of those treated before 2017 and a no-drain policy (NDP) group comprising patients who underwent LAR after 2017.

Eligible criteria included histologically proven rectal adenocarcinoma located 12 cm or less from the anal verge, no evidence of distance metastases, elective laparoscopic or robotic LAR, and age above 18 years old.

The following data were retrospectively extracted from prospectively collected clinical records: age, gender, body max index (BMI), Charlson Comorbidity Index [20], ECOG performance status [21], preoperative treatment (chemotherapy ± radiotherapy), ASA score, stage, preoperative albumin, and hematocrit values (retrieved typically 2 or 3 weeks before surgery), tumor distance from anal verge, operative time and blood loss volume, anastomosis method (stapled or hand-sewn), type of surgery (laparoscopic/open), temporary diversion (ileostomy or colostomy), AL, length of stay (LOS), and postoperative complications, defined as any clinical event who would characterize the postoperative stay and delay hospital discharge, reported using the Clavien-Dindo classification [22]. A grade equal to or greater than IIIa was considered major complication. Ileus was defined as inability to tolerate a diet for more than 3 days or need of naso-gastric tube insertion.

AL was suspected in case of peritonitis, septic symptoms, and fecal or purulent discharge from AD, if present. Upon suspicion, patients underwent a radiological examination with contrast-enhanced computed tomography (CT) scan. AL was defined and classified according to the International Study Group of Rectal Cancer [23].

All patients were followed for at least 90 days after surgery. The rate of AL and related interventions was considered the primary endpoint of the study. Secondary endpoints included postoperative complications, LOS, readmission, temporary diversion rate, and surgeons’ adherence to the no-drain policy.

Data on patients requiring treatment for AL were collected to investigate differences between treatment for leak in patients with and without drainage, as well as between DP and NDP groups.

Before the operation, all patients underwent a preoperative evaluation, which included colonoscopy with biopsy, chest and abdomen CT scan, pelvic magnetic resonance imaging (MRI), surgical examination, and multidisciplinary team discussion. A multidisciplinary assessment, following international guidelines, was performed for all patients [24].

All patients underwent preoperative bowel preparation and received prophylactic antibiotics, consisting of Macrogol 16 sachet in 4 l of water and paromomycin 250 mg 2 tablets 3 times the day before surgery. Both laparoscopic and robotic LAR procedures were performed, involving high ligation of the inferior mesenteric artery and vein, mobilization of the left colon, and total mesorectal excision preserving the hypogastric nerves. Anastomosis was either hand-sewn or created with a mechanical stapler. A defunctioning stoma was fashioned, and an abdominal drainage was placed at the discretion of the surgeon. Postoperative management was standardized for all patients. In cases where drainage was placed, the volume and nature of fluid were evaluated daily. Drain removal was performed when the output was less than 100 ml in 24 h.

Statistical analyses were conducted using JMP Pro 15.0 (SAS Institute Inc., Cary, NC, USA). Continuous data were presented as means ± standard deviation (SD) and analyzed using a *t*-test if normally distributed. Non-normally distributed continuous data were expressed as medians and interquartile ranges (IQRs) and analyzed using the Mann–Whitney *U* test. Categorical and ordinal data were presented as counts and percentages. Categorical data were analyzed using the *χ*^2^ test or Fisher’s exact test, while ordinal data were analyzed using the Mann–Whitney *U* test. All analyses were two-sided, and *p*-values ≤ 0.05 were considered statistically significant.

## Results

Among the 272 included patients, 188 (69.1%) were in the NDP group, while 84 (30.9%) were in the DP group. Table 1 summarizes the baseline preoperative characteristics, which were similar between the two groups, including demographics, tumor stage, and neoadjuvant treatment.

**Table 1:** Preoperative characteristics

	NDP *n* = 188	DP *n* = 84	*p*
Age †	63.1 (12.4)	65.9 (12.1)	0.082
Male	111 (59.0%)	53 (63.1%)	0.526
BMI (kg/m^2^) †	25.1 (4.4)	24.7 (3.4)	0.369
CCI †	4.6 (1.7)	5.02 (2.2)	0.098
ECOG-PS 0 1 2	138 (73.4%) 45 (23.9%) 5 (2.7%)	58 (69.0%) 25 (29.8%) 1 (1.2%)	0.514
ASA 1 2 3	36 (19.1%) 127 (67.5%) 25 (13.3%)	14 (16.7%) 54 (64.3%) 16 (19.0%)	0.469
S-Albumin g/dl †	41.4 (3.7)	41.4 (3.6)	0.988
Hematocrit (%)†	40.5 (4.5)	38.9 (5.1)	0.028*
Creatinine (mg/dl) †	0.81 (0.2)	0.84 (0.2)	0.478
Neoadjuvant therapy	105 (55.6%)	53 (63.1%)	0.261
Prior abdominal surgery	66 (35.1%)	38 (45.2%)	0.114
Tumor distance from anal verge (cm) †	7.4 (3.1)	7.3 (2.8)	0.784
Stage 0 1 2 3 4	15 (8.0%) 86 (45.7%) 31 (16.5%) 46 (24.5%) 10 (5.3%)	12 (14.3%) 24 (28.6%) 21 (25%) 22 (26.2%) 5 (6.0%)	0.057

*ASA*, American Society of Anesthesiologist Score; *BMI*, body mass index; *CCI*, Charlson Comorbidity Index; *ECOG*, Eastern Cooperative Oncology Group Performance Status

*Statistically significant

†Mean (standard deviation)

Table 2 describes intraoperative and postoperative characteristics. Patients in the NDP group were less likely to receive an AD (16.5% NDP vs 76.2% DP, *p* < 0.001). The NDP group also had a lower percentage of patients with temporary diversion (83.5% vs 95.2%, *p* = 0.004) and a higher anastomosis median distance from anal verge (4 cm (IQR 3–7) vs 3 cm (IQR 2–5), *p* = 0.031). The operative time was longer in the DP group (254.5 min vs 309.3 min, *p* < 0.001). Both groups had similar rates of postoperative complications, LOS, and readmission at 90 days.

**Table 2:** Intra- and postoperative characteristics

	NDP *n* = 188	DP *n* = 84	*p*
Drain placement	31 (16.5%)	64 (76.2%)	< 0.001*
Conversion to open	0 (0%)	2 (2.4%)	0.095
Operative time (min) †	254.5 (66.0)	309.3 (67.2)	< 0.001*
Temporary diversion	157 (83.5%)	80 (95.2%)	0.004*
Anastomosis distance from anal verge (cm) Median (IQR)	4 (3–7)	3 (2–5)	0.031*
Clavien-Dindo 0 I II IIIa IIIb IV	143 (76.1%) 4 (2.1%) 16 (8.5%) 9 (4.8%) 12 (6.4%) 4 (2.1%)	63 (75.0%) 3 (3.6%) 9 (4.8%) 2 (2.4%) 5 (5.9%) 2 (2.4%)	0.883
Anastomotic leak	21 (11.2%)	9 (10.7%)	1.000
Anastomotic leak grade A B C	4 (19.1%) 6 (28.6%) 11 (52.4%)	2 (28.6%) 1 (11.1%) 6 (66.7%)	0.759
Days to diagnosis of leak Median (IQR)	15 (2–22.5)	6 (3.5–22.5)	0.981
Complications other than leak	34 (18.1%)	15 (17.9%)	0.963
Ileus	10 (5.3%)	4 (4.8%)	0.847
Length of stay (days)°	5 (4–7)	6 (4.3–8)	0.059
Readmission < 90 days	5 (2.7%)	3 (3.6%)	0.706

*Statistically significant

°Mann–Whitney *U* test

†Mean (standard deviation)

*IQR*, interquartile range

When inserted, ADs were removed earlier in the NDP group compared to the DP group (2 days (IQR 2–3) vs 3.5 days (IQR 2–7), *p* < 0.001; Table 3).

**Table 3:** Days of maintaining a drain according to group

	Total	NDP	DP	
Days drain maintained Median (IQR)	3 (2–4)	2 (2–3)	3.5 (2–7)	< 0.001*

*Statistically significant

*IQR*, interquartile range

**Fig. 1:**
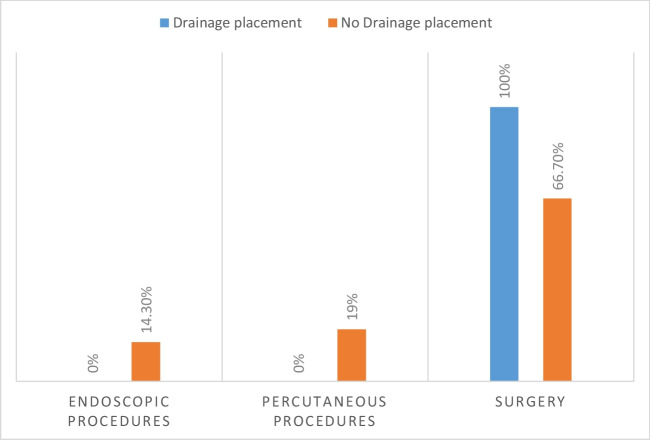
Type of treatment for leak with or without drain placement

The AL rate (11.2% NDP vs 10.7% DP, *p* = 1.000) and grade (grade A with 19.1% vs 28.6%; grade B with 28.6% vs 11.1%; grade C with 52.4% vs 66.7%, *p* = 0.759) did not significantly differ between groups. All patients with symptomatic AL and AD underwent surgery as treatment for leak, while those without AD were treated with surgery (66.7%), endoscopic (19.0%), and percutaneous (14.3%) procedures (Fig. 1).

All patients diagnosed with a leak received an ileostomy at primary surgery. Median length of stay for patients who developed a leak was 12.5 days (IQR 7–22), significantly longer compared to those who did not develop an anastomotic leak (5 days (IQR 5–7), *p* < 0.001). Seventeen patients underwent surgery for symptomatic leaks regardless of the policy, with a median hospital stay of 17 days (IQR 11–34). Although not statistically significant, this was longer compared to the seven patients who were treated for symptomatic leaks with endoscopic or percutaneous procedures, who had a median stay of 7 days (IQR 6–13) (*p* = 0.08).

**Fig. 2:**
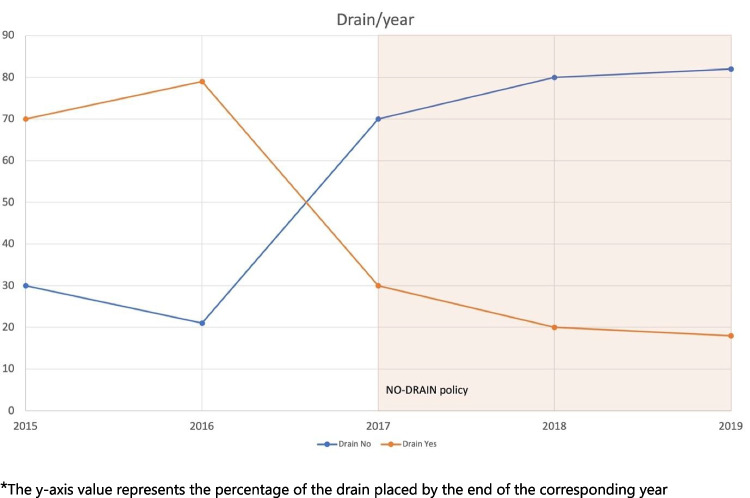
Cases/drain according to year. The *y*-axis value represents the percentage of the drain placed by the end of the corresponding year

Figure 2 reports the trend of drain placement throughout the study years. Three years after implementing the no-drain policy, AD was utilized in 16.5% of cases, compared to 76.2% at the study’s outset (risk ratio for drain placement across the two groups: 0.22, 95% CI 0.15 to 0.31, *p* < 0.0001). The percentage of AD placed changed significantly in 2017 after the implementation of the no-drain policy.

## Discussion

Results of this study indicate that the no-drain policy did not significantly impact the rate of AL with no difference in postoperative complications rate.

Despite the implementation of the no-drain policy for 3 years, perianastomotic drains were still utilized in nearly 20% of cases.

Various reasons have historically led surgeons to place an AD after rectal surgery. ADs were believed to facilitate rapid detection and mitigate the consequences of AL by draining infected material. However, Urbach et al. [10] demonstrated in a meta-analysis that AD placement in colonic and rectal surgery did not reduce the incidence of AL or its repercussions. Their study found that only 5% of AL cases was diagnosed because of purulent or fecal discharge from the drain. This finding was supported by Denost et al. [12] who described the time discrepancy between AL diagnosis and drain removal in their multicenter randomized trial assessing the effect of AD placement after rectal cancer surgery. They reported a median time of diagnosis of pelvic sepsis of 7.8 days after surgery, while median drain removal happened at 5.5 postoperative days. In our study, the median diagnosis of AL was 15 days after surgery (IQR 2–22.5) for the NDP group and 6 days after surgery (IQR 3.5–22.5) for the DP group (*p* = 0.981), with AD removal occurring at a median of 2 days after surgery in the NDP group compared to 3.5 days after surgery in the DP group (*p* < 0.001). This conclusion raises doubts about the usefulness of AD in diagnosing AL.

Another hypothesis suggests that rectal dissection with TME results in a large raw space in the pelvis and around the perianastomotic area. This, combined with previous neoadjuvant radiotherapy, intraoperative lavage, and the absence of serosa at the AL site, may promote bacterial overgrowth and the formation of bacterial metalloproteinases that could endanger weakness points of the colorectal anastomosis [25]. However, several studies have discredited this theory by demonstrating no reduction in the incidence of AL when drains are used to remove clot, debris, and perianastomotic fluid [10, 26, 27] .

A recent meta-analysis of four randomized clinical trials (RCTs) did not support the use of AD following colorectal anastomosis. This analysis demonstrated that AD placement did not affect the rate of AL, mortality, pelvic sepsis, wound infections, and reintervention for abdominal complications compared to patients for whom AD was not utilized [15].

Another meta-analysis [28] including three RCTs and five non-RCTs investigated the rate of extraperitoneal AL rate in patients with or without AD. The results initially suggested a lower rate of extraperitoneal AL and reintervention in patients with AD compared to those without AD. However, this result difference was not retained after a subgroup analysis limited to RCTs, indicating no significant difference between the two groups.

The COMPASS (COMPlicAted intra-abdominal collectionS after colorectal Surgery) study further demonstrated the limited efficacy of intraperitoneal drains in elective colorectal surgery [29]. A total of 1805 consecutive patients underwent elective colorectal surgery across 22 countries and were prospectively enrolled in the study. The findings revealed that drainage placement did not result in an early diagnosis of intra-abdominal collections. Additionally, patients who had drainage placed had an increased risk of surgical site infection and experienced delayed hospital discharge. However, it is worth noting that the study considered all colorectal procedures without subclassifying the risk according to the type of surgery performed.

In our study, we observed a higher rate of surgery after AL in patients with an AD. Specifically, all patients with a symptomatic AL (grade higher than A) and an AD underwent surgery, including two cases treated with peritoneal lavage, two with leak suture, and one with anastomosis resection. Conversely, patients without a drain who experienced an AL grade B or C were treated with surgery (66.7%), endoscopic (19.0%), and percutaneous (14.3%) procedures (Fig. 1). One explanation is that percutaneous and endoscopic approaches have been recently implemented as valid less invasive alternatives to surgery, particularly for patients without severe clinical conditions.

Even when strongly suggested by robust evidence, introducing a new routine of practice may take time and effort. The surgical community, especially, is somehow resistant to leave old paradigms which are thought to be safer even if never fully demonstrated in a scientific way. It took years and still encounters resistance to the application of ERAS® protocols which undoubtedly improved our practice towards a more evidence-based surgical perioperative care [30]. For instance, ERAS® guidelines advise against the routine use of an AD after colorectal surgery. Throughout the study period of our investigation, surgeons were left with the choice of whether to utilize or not an AD. In fact, surgeons’ perception in assessing the risk of AL remains one of the main parameters in the absence of a consolidated tool for AL risk assessment. The present analysis performed outside a RCT may provide new insights in the adoption of such a controversial habit as routine drain use. In the present study, patients in the DP group experienced longer operative times, a lower anastomosis distance from the anal verge (although possibly not clinically relevant), and a higher rate of ostomy. However, these differences did not result in worse clinical outcomes, as the rates of AL, complications, and LOS were similar between groups.

Generally based on hazard assessment, AD was placed when “hard and complicated” surgeries took place. In our study, before the introduction of a no-drain policy, 20 to 30% of cases were already performed without the use of an AD. This could assess a pre-existent propensity towards a no-drain routine. After implementing a structured no-drain policy, the adoption was quick from the first year with a steep incline in no AD cases, assessing towards an 80% policy adoption at the end of the study period (Fig. 2). Moreover, among patients who received an AD in the NDP group, the drain was removed earlier compared to the DP group, suggesting that implementing a no-drain policy may lead to faster drain removal.

Our study has some important limitations. Firstly, it is a retrospective analysis, although data were sourced from a prospectively maintained database. Consequently, some variables may be influenced by unknown confounders. Additionally, the implementation of the no-drain policy in the later years was not mandatory; surgeons retained the discretion to decide whether to use an AD, introducing another potential confounding factor. Furthermore, this study was conducted at only two high-volume tertiary referral colorectal departments in Italy, limiting the generalizability and reproducibility of the results to other settings.

In conclusion, the introduction of a no-drain policy received a good adoption rate and did not affect negatively the surgical outcomes.

## Supplementary Information

Below is the link to the electronic supplementary material.

Supplementary file1 (DOCX 33 KB)

## Data Availability

Data are stored in a secured database and would be available upon request to jacopocrippamd@gmail.com.
